# 2012 – The beginning of a new world for Digital Pathology

**DOI:** 10.1186/1746-1596-8-S1-S1

**Published:** 2013-09-30

**Authors:** Vincenzo Della Mea, Roberto Mencarelli

**Affiliations:** 1 Dept. of Mathematics and Computer Science, University of Udine, Italy; 2 Clinical Pathology Department, Local Healthcare Authority, Rovigo, Italy

## 

The series of the biennial European congresses on Telepathology reached with this Venice edition its 11th appointment: more than 20 years of research in a continuously growing field. It is also the fifth time the congress brings the additional name of International Congress of Virtual Microscopy: eight years ago the International Academy of Telepathology decided to recognize this way the importance of virtual microscopy, also called Whole Slide Imaging, in the renovation of the telepathology scenario. In the last congress the International Academy of Digital Pathology was also founded, to replace and continue the great work of the International Academy of Telepathology that organized most of this congress series. This step recollects under the single term “digital pathology” all the technologies that are tranforming the traditional, “analog” pathologist in a digital professional that work on digitized slides, with software tools that enable to better exploit his/her knowledge, leaving routine details to the computers and networks. Once slides are digitized, a whole bunch of applications come natural and available: telediagnosis, teleconsultation, e-learning, long term storage, up to image analysis, with the forthcoming field of digital immunohistochemistry. No need to carry out additional operations: slides are ready for further digital treatment, present and future.

Digital Pathology is made possible not only thanks to the research grown in the last many years and presented at every Congress of this series. Every year the processors power increases, memory becomes larger and larger, and thus computers become more and more adequate to such large, information-filled objects that are the so-called digital slides.

This 2012 Congress hosted more than 80 presentations from 23 countries of the world. It represents the current state of the art in the field of digital pathology: what can be read in the present proceedings will represent the future of Pathology in the short, mid and even long term, thus providing insights on a new world which the traditional and crucial work of the pathologist should remain under his/her own control, made easier and more productive through digital tools.

